# Dietary Risk of Neonicotinoids and Other Pesticides in Mexican Children Residing in Agroindustrial Zones

**DOI:** 10.3390/ijerph23081009

**Published:** 2026-08-01

**Authors:** Irma Aidé García Villegas, Silvia Lizette Ramos de Robles, Claudia Alvarado Osuna

**Affiliations:** 1 Departamento de Ciencias Ambientales, Centro Universitario de Ciencias Biológicas y Agropecuarias, Universidad de Guadalajara, Zapopan 44600, Mexico; irma.garcia0963@alumnos.udg.mx; 2 Unidad de Tecnología Alimentaria, Centro de Investigación y Asistencia en Tecnología y Diseño del Estado de Jalisco, Zapopan 45019, Mexico; calvarado@ciatej.mx

**Keywords:** pesticides, neonicotinoids, dietary health risk, preschool children

## Abstract

**Highlights:**

**Public health relevance—How does this work relate to a public health issue?**

- Children aged 4–5 years in the agroindustrial zone of Jalisco are repeatedly exposed to multiple pesticide residues through their diet. 87% of analyzed food samples contained at least one detectable compound, and 74% had two or more residues simultaneously.
- Five neonicotinoids were identified in staple foods, with imidacloprid being the most frequently detected (61.3%), and guava and tomato as the main sources of exposure.

**Public health significance—Why is this work of significance to public health?**

- Children could have been exposed to 34 pesticides through their habitual diet, with raw food consumption accounting for 96% of the total estimated weekly intake.
- Seven samples exceeded the Maximum Residue Limits (MRLs) established by the EU, the US, or Mexico. The detection of carbendazim—a probable human carcinogen banned in the US—in strawberry, apple, and tomato highlights a regulatory asymmetry that represents a critical gap in the protection of vulnerable populations.

**Public health implications—What are the key implications or messages for practitioners, policymakers and/or researchers in public health?**

- Policymakers should harmonize MRLs across jurisdictions, regulate pesticide-commodity combinations currently unregulated in Mexico, and strengthen monitoring programs. The continued authorization of neonicotinoids in Mexico—despite bans or restrictions in the European Union and other countries on grounds of human health and ecosystem harm—warrants urgent regulatory reassessment.
- Researchers must develop mixture risk assessment approaches that account for additive, synergistic, or potentiating effects, and conduct longitudinal studies on the neurodevelopmental, endocrine, and immunological consequences of chronic multi-pesticide dietary exposure (DE) in children from agroindustrial zones.

**Abstract:**

Dietary pesticide exposure is a growing public health concern, particularly for children in agricultural regions. This study assessed the occurrence and concentration of neonicotinoids and other pesticide residues in foods frequently consumed by preschool children (4–5 years) in an agroindustrial zone of Jalisco, Mexico, and estimated their weekly dietary exposure (DE). Thirty-one food samples representing 18 items were collected from retail sites identified through seven-day dietary records completed by 23 families. Samples were analyzed by LC/ESI-MS/MS covering 236 compounds; exposure was estimated considering raw and cooked consumption patterns and processing factors. Residues were detected in 87% of samples, yielding 34 distinct compounds, including 5 neonicotinoids; imidacloprid was the most frequently detected. Strawberry, raspberry, apple, grape, and tomato showed the highest residue loads; seven samples exceeded MRLs set by the EU, USA, or Mexico. Raw food consumption accounted for 96% of total estimated exposure, with pyrimethanil, boscalid, propamocarb, and imidacloprid as principal contributors. These findings confirm that children in this population face repeated, simultaneous exposure to multiple pesticides through diet, underscoring the need for stronger food safety monitoring, harmonized regulatory limits, and cumulative risk assessment frameworks for vulnerable populations.

## 1. Introduction

Pesticide use in food production has increased markedly in recent decades. According to Tudi et al. [1], approximately one-third of global agricultural output relies on pesticide application. According to the FAOSTAT database [2], a total of 3,697,552 tonnes of pesticide inputs were applied worldwide in 2022. Consequently, the detection of pesticide residues in agricultural commodities has become increasingly frequent [3,4].

While ensuring food security for the global population remains the primary justification for pesticide use, its application has become increasingly contested given the well-documented impacts on ecosystems and human health. Epidemiological and toxicological evidence has established associations between pesticide exposure and a broad range of adverse health outcomes, including various forms of cancer, allergic responses, and disruption of the nervous, reproductive, and endocrine systems, as well as congenital malformations [5].

Mexico allocates 24.6 million hectares to agricultural production, ranking 11th globally in crop output and 8th in food exports. The state of Jalisco, located in western Mexico, is the leading agricultural region, contributing approximately 25% of the nation’s food supply. However, this agricultural productivity is embedded within an agroindustrial model characterized by intensive pesticide use, which has demonstrated measurable ecological impacts and potential risks to human health [6].

Within this context, neonicotinoids are of particular concern. This class of systemic insecticides became the most widely used insecticide group worldwide within a few decades of its introduction [7,8]. Initially introduced as an alternative considered *less toxic* to mammals and *safe* for vertebrates, and celebrated for their high efficacy in pest control [9], neonicotinoids have since been shown to exert significant adverse effects on both ecosystems and human health.

Neonicotinoids act by binding irreversibly to nicotinic acetylcholine receptors (nAChRs) in the insect central nervous system. Evidence has accumulated demonstrating collateral effects on non-target organisms, including pollinators, aquatic and terrestrial invertebrates, birds, mammals, and fish [10,11]. Among pollinators, large-scale colony losses in honey bees (*Apis mellifera*) have been associated with neonicotinoid exposure [12,13], along with sublethal effects including reduced longevity, immunosuppression, and impaired learning and memory [14,15]. In Lepidoptera, neonicotinoid exposure has been linked to reduced larval size and mortality in monarch butterflies [16]. In avian species, particularly Galliformes and Columbiformes with seed-based diets, population declines and acute mortality have been documented [17,18]. Adverse effects on aquatic fauna have also been reported, including population declines in smelt fish [19].

Regarding human health, neonicotinoids have been associated with a range of potential toxic effects, including reproductive toxicity, neurotoxicity, hepatotoxicity, hepatocarcinogenicity, immunotoxicity, and genotoxicity [20]. They have been identified as endocrine-disrupting compounds capable of inducing apoptosis and histopathological alterations in prostatic epithelial cells [21], impairing reproductive function and hormonal regulation [22]. Genotoxic effects have been directly demonstrated in human cells: in vitro exposure of human peripheral blood lymphocytes to imidacloprid induced dose-dependent DNA damage, along with increased micronuclei and sister chromatid exchange frequencies [23]. Beyond these mechanistic effects, aggregate dietary risk assessments using an imidacloprid-equivalency approach have documented cumulative neonicotinoid exposure among school-age children through fruit and vegetable consumption, underscoring the relevance of cumulative rather than single-compound exposure metrics in pediatric populations [24].

On the basis of these considerations, the present study was conducted to assess the occurrence and concentration of neonicotinoids and other pesticides in foods frequently consumed by 4- and 5-year-old children and to characterize the potential dietary health risks. The focus on a pediatric population is justified by the heightened vulnerability of children, whose physiological characteristics, behavioral patterns, and developmental stage render them disproportionately susceptible to pesticide exposure compared to adults [5].

Although the study was designed primarily around neonicotinoids, the analytical method employed enabled the simultaneous screening of 236 pesticide compounds, including seven neonicotinoid insecticides (acetamiprid, clothianidin, imidacloprid, dinotefuran, nitenpyram, thiacloprid, and thiamethoxam). The detection of additional pesticide residues in the food samples is therefore also reported, providing evidence of concurrent multi-compound exposure.

Although neonicotinoids accounted for only 5 of the 34 pesticide compounds detected in this study, and non-neonicotinoid fungicides such as pyrimethanil and boscalid contributed more substantially to the overall estimated dietary exposure, this insecticide class was deliberately prioritized for several reasons. First, neonicotinoids represent a regulatory paradox in Mexico: several compounds within this class are banned or restricted in the European Union and other jurisdictions on the basis of established ecotoxicological and human health concerns, yet remain fully authorized for agricultural use in Mexico, including in the study region. Second, unlike most of the non-neonicotinoid fungicides detected, neonicotinoids are systemic, water-soluble insecticides that cannot be removed by washing or peeling and exhibit marked thermostability during cooking, properties that render them a persistent and largely unavoidable route of dietary exposure in young children. Third, to the authors’ knowledge, no prior study has characterized dietary neonicotinoid exposure specifically among preschool-aged children in an agroindustrial region of Mexico, representing an important evidence gap given the documented neurodevelopmental and endocrine vulnerability of this age group. For these reasons, this manuscript retains a dual framing—combining a focused analysis of neonicotinoid residues with a broader characterization of concurrent multi-pesticide dietary exposure—rather than treating neonicotinoids as the sole object of study.

## 2. Materials and Methods

### 2.1. Study Population and Dietary Data Collection

An open invitation was extended to mothers of children enrolled at a preschool located in San Andrés Ixtlan, Jalisco, Mexico. A total of 23 mothers whose children were between 4 and 5 years of age agreed to participate. Mothers were trained to complete standardized dietary records capturing their children’s food intake over seven consecutive days (Monday through Sunday). Records included the identity and quantity of all foods consumed, time of consumption, preparation method, and the retail locations where foods were purchased (local stores, market stalls, and street vendors).

### 2.2. Food Sample Collection

The most frequently consumed foods were identified from the dietary records and subsequently purchased from the same retail locations reported by the study participants, with a minimum of two purchase sites covered per food item. A total of five purchase sites were identified across the municipalities of San Andrés Ixtlán and Ciudad Guzmán, Jalisco. In total, 31 samples representing 18 distinct food items were collected, placed in clearly labeled resealable polyethylene bags, stored in coolers, and subsequently refrigerated until dispatch to the analytical laboratory.

The sampled food groups and items were as follows: Cereals (5 samples): white and yellow maize tortillas and corn on the cob. Fruits (13 samples): raspberry, strawberry, apple, orange, banana, guava, mandarin, and green grape. Vegetables (9 samples): avocado, onion, chayote, tomato, and cucumber. Tubers (2 samples): potato. Legumes (2 samples): common bean.

### 2.3. Analytical Methods for Food Sample Analysis

All food samples were analyzed at the Agriculture and Food Laboratory (AFL) of the University of Guelph, Canada, which holds accreditation from the Canadian Association for Laboratory Accreditation (CALA) and the Standards Council of Canada (SCC), and operates in compliance with ISO/IEC 17025 standards [25]. Analyses were performed by liquid chromatography coupled to electrospray ionization tandem mass spectrometry (LC/ESI-MS/MS), a validated method for pesticide determination in food matrices [26]. The analytical protocol covered 236 pesticide compounds, including fipronil and the neonicotinoids acetamiprid, clothianidin, imidacloprid, dinotefuran, thiacloprid, and thiamethoxam.

Pesticide residues were extracted using the QuEChERS (quick, easy, cheap, effective, rugged, and safe) method. Briefly, a representative sample was extracted into a solution of 1% acetic acid (CH_3_COOH) in acetonitrile in the presence of anhydrous sodium acetate (C_2_H_3_NaO_2_) and magnesium sulfate (MgSO_4_). Cleanup of the supernatant was performed by dispersive solid-phase extraction (dispersive-SPE) using MgSO_4_ and primary–secondary amine (PSA) exchange material. The concentrated extract was quantified by LC-MS/MS using matrix-matched standard calibration curves and isotopically labeled internal standards.

### 2.4. Dietary Exposure Estimation

Weekly dietary exposure to pesticide residues was estimated using the following formula [27]:

*Dietary Exposure = Σ (Concentration of chemical in food × Food consumption)*

The pesticide concentration term in the above formula incorporated two components: (a) the analytical result obtained for each compound in the food sample; and (b) the form of consumption as documented in the dietary records. For component (a), mean pesticide residue concentrations were used; however, for compounds detected below the limit of quantification (<MQL) or below the limit of detection (<MDL), two bounding scenarios were applied: a lower-bound scenario, in which values below detection or quantification thresholds were set to zero, and an upper-bound scenario, in which such values were assigned the MDL or MQL of the respective pesticide [28,29,30].

DE estimates accounted for the effects of food processing and thermal preparation practices. Processing factors (PF), based on internationally recognized values (Table 1), were applied to adjust pesticide residue concentrations in cooked foods. When a PF was unavailable for a specific pesticide–food combination, a surrogate PF was selected using one of two criteria: (i) a pesticide belonging to the same chemical class and exhibiting similar relevant physicochemical properties, or (ii) the same pesticide in a food matrix with comparable characteristics subjected to an equivalent thermal process [31,32,33].

Final pesticide concentrations in processed foods were calculated following the equation of Hrynko et al. [34].

*C*processed (mg kg^−1^) = PF × *C*raw (mg kg^−1^)

where PF is the processing factor, C*processed* is the pesticide concentration in the thermally processed food item, and C*raw* is the pesticide concentration in the unprocessed commodity as reported by the laboratory.

**Table 1:** Processing factors (PF) of pesticides according to food matrix, and type of thermal process used.

Pesticide	Class/Chemical Group	Food Matrix	Thermal Process	PF	Reference/ Food Matrix ^†^
Boscalid	Pyridine-Carboxamide	tomato	boiled	0.03	[35]/tomato
Fluoxastrobin (Azoxystrobin used instead)	Dihydro-dioxazin	bean, potato	cooked	0.3 *	[33]/bean
Pyrimethanil	Anilino-Pyrimidine	banana	cooked	1.0	[33]/peas
Pyraclostrobin	Methoxy-carbamate	tomato	cooked	0.79	[33]/spinach
Carbendazim	Benzimidazole	tomato, onion, chayote, corn on cob, bean	baking	0.8	[36]/cereals
Imidacloprid	Neonicotinoid	tomato, potato, chayote, onion, corn on cob	cooked/ boiled	0.58	[32]/ potato, celery, turnip
Acetamiprid	Neonicotinoid	tomato	fried	0.99	[34]/apple
Thiamethoxam	Neonicotinoid	tomato	cooked/ boiled	0.58	[32] potato, celery, turnip
Dinotefuran	Neonicotinoid	tomato, onion	boiled	0.4	[37]/rice
Clothianidin	Neonicotinoid	tomato, corn on cob	cooked/ boiled	0.58	[32]/ potato, celery, turnip
Methomyl	Carbamate	corn	cooked/ boiled	0.17	[32]/pumpkin
Methoxifenozide	Diacylhydracine	corn	cooked/ boiled	0.4	[32,35]/ lentils, cereals/apples
Novaluron (Triflumuron used instead)	Benzoylurea	corn, tomato	cooked	<0.13 *	[38]/peaches
Spirodiclofen	Tetramic and tetronic acids	chayote	cooked	<0.47	[33]/strawberry
Spirotetramat (Spiromesifen used instead)	Tetramic and tetronic acids	chayote	cooked	0.65 *	[33]/strawberry
Mandipropamid	Mandelic acid amide	chayote	fried	0.09	[37]/potato

^†^ Food matrix reported in the reference. * Processing factors from different pesticide with similar mechanism of action.

The pesticides detected were classified according to their type of use, chemical family/group and chemical name. Residue concentrations were also compared according to three benchmarks to estimate health risk.

### 2.5. Ethical Implications

Participation in this study was voluntarily requested from the mothers of preschool children enrolled at a public preschool in San Andrés Ixtlán, Jalisco, Mexico. Prior to enrollment, mothers were informed of the study’s objectives, procedures, and voluntary nature, and all provided written informed consent. Their participation involved completing a structured seven-day dietary logbook in which they recorded, for each of their children’s meals, the foods consumed, the quantities ingested, the form of processing or preparation (raw, cooked, boiled, fried, etc.), and the retail location where each food item was purchased. Food samples used for pesticide analysis were purchased separately from the retail sites identified through these records. No biological samples or invasive procedures were carried out on the children. All information collected was treated confidentially and used exclusively for research purposes associated with a Master’s thesis and its derived publications, in accordance with the ethical principles for research involving human participants set out in the Declaration of Helsinki and its later amendments.

## 3. Results

### 3.1. Pesticide Residues Detected in Food Samples

A total of 34 pesticide compounds were detected across the 31 food samples analyzed, including 5 neonicotinoids. Of these, 22 compounds were quantifiable at concentrations above their respective limits of quantification, while 12 were detected below the limit of quantification and reported under both bounding scenarios (as zero under lower-bound conditions and as the MQL value under upper-bound conditions).

**Figure 1:**
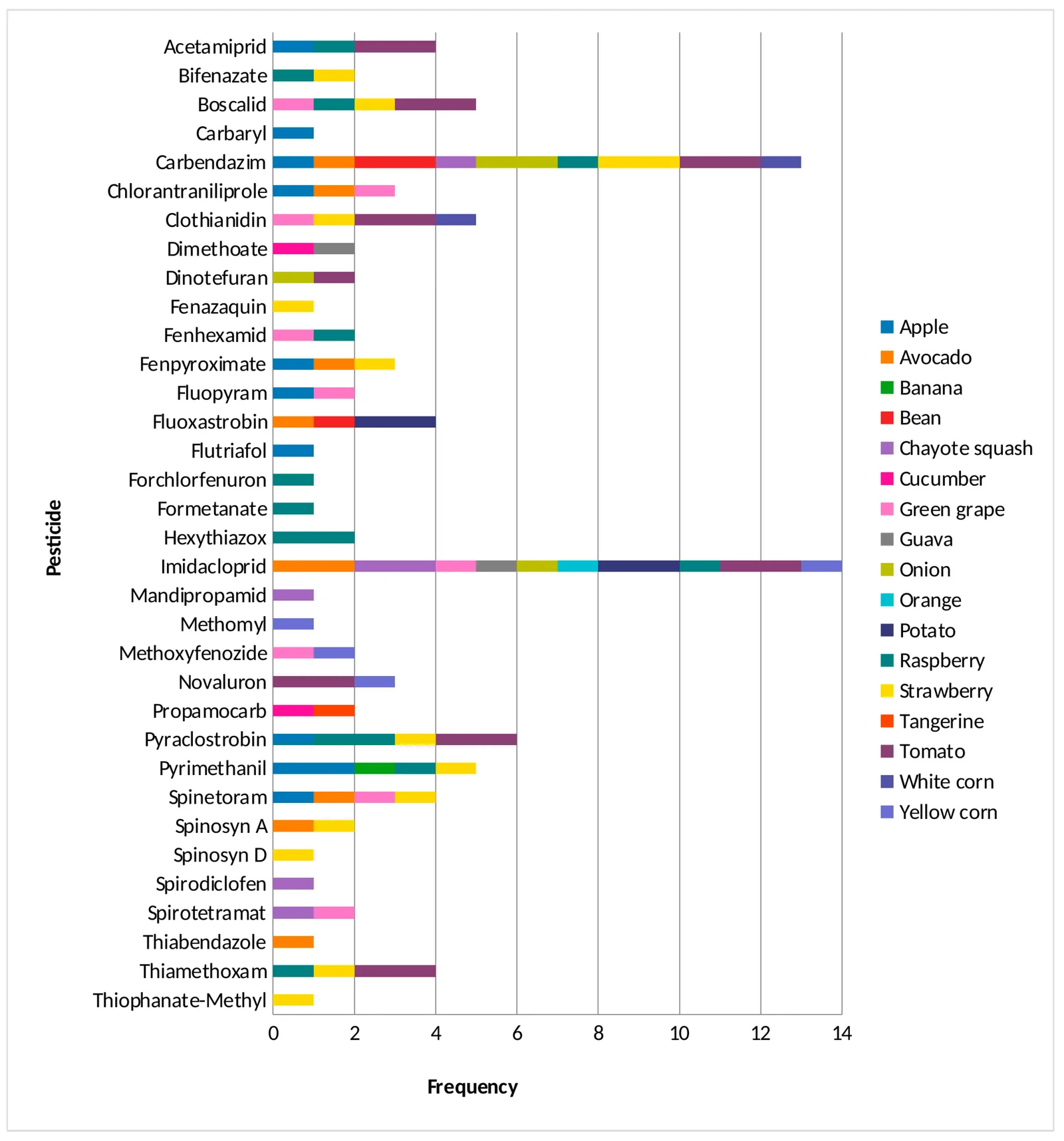
Frequency of occurrence of individual pesticides across food samples analyzed.

The results are presented considering the quantification of pesticides at their quantifiable and non-quantifiable concentrations (<MDL, <MQL). The detection frequency of each individual pesticide is shown in Figure 1, where imidacloprid was the most frequently detected pesticide, followed by carbendazim and pyraclostrobin.

**Figure 2:**
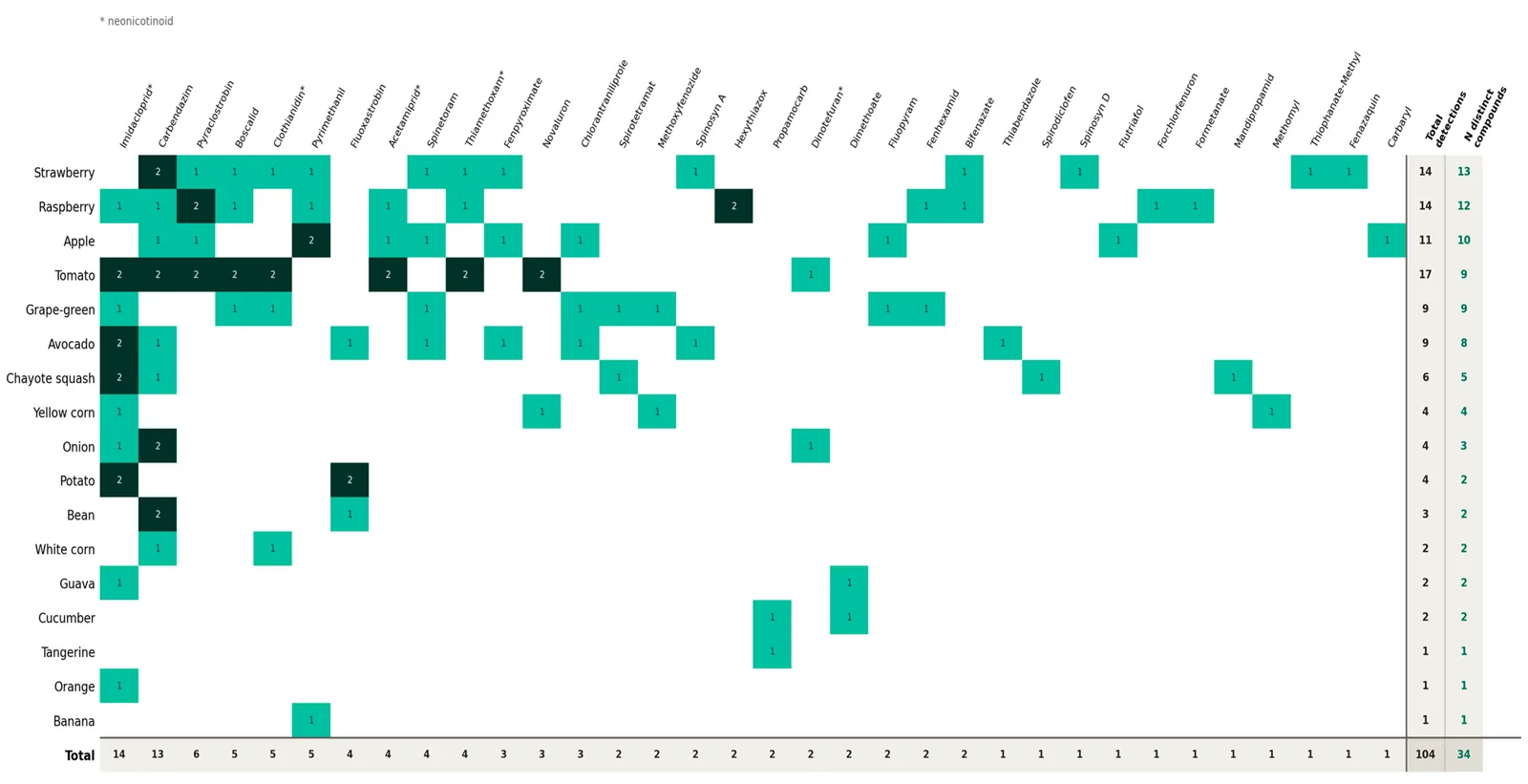
Frequency of pesticide detection by food matrix. Cell values indicate the number of positive samples for each pesticide–food combination. Row totals show the total number of detections and the number of distinct pesticide compounds per food item; column totals show the total detections per pesticide across all foods. Asterisks denote neonicotinoid compounds.

Pesticide residues were detected in 87% of the samples analyzed. Of the pesticide-positive samples, 74% contained two or more distinct pesticide compounds simultaneously. Figure 2 shows the frequency of pesticide detections per food item; tomato, strawberry, and raspberry contained up to 10 pesticides. The highest numbers of pesticide residues were recorded in strawberry (13 compounds), raspberry (12), apple (10), grape (9), and tomato (9). Maize tortilla was the only food commodity in which no pesticide traces were detected.

Classification of the most frequently detected pesticides revealed that insecticides and acaricides—including neonicotinoids—constituted the predominant chemical group in the food samples, followed by fungicides and plant growth regulators. Of the 31 food samples analyzed, 61.3% contained at least one neonicotinoid residue: 14 samples contained one neonicotinoid, three contained two, one contained four, and one contained five distinct neonicotinoid compounds. Tomato was the commodity with the greatest number of neonicotinoid detections, with up to four and five different neonicotinoids per sample.

A total of seven food samples exceeded Maximum Residue Limits (MRLs) established by the European Union (EU), the United States (US), or Mexico (Table 2), corresponding to nine individual pesticide–MRL exceedances. It is noteworthy that MRLs are not harmonized across jurisdictions, and that Mexico has not established MRLs for certain pesticide–commodity combinations, such as carbendazim in strawberry and apple or imidacloprid in guava. Of these nine exceedances, four involved EU MRLs, one involved Mexican MRLs, and four involved US regulatory thresholds, where carbendazim holds a zero tolerance. This total of nine exceedances across seven samples reflects the fact that some samples exceeded the MRL for more than one pesticide simultaneously: two strawberry samples each exceeded the MRL for carbendazim together with either boscalid or thiophanate-methyl, and are therefore counted twice among the nine exceedances shown in Table 2.

### 3.2. Weekly Dietary Exposure Estimation

Based on the dietary records of the 23 children enrolled in the study, consumption ranges were obtained for each analyzed food item, distinguishing between raw and cooked consumption modes. Fruits were predominantly consumed raw, vegetables showed a mixed raw/cooked pattern, and tubers and legumes were consumed exclusively cooked (Table 3).

Considering the dietary patterns of the study population, the quantities consumed, thermal processing practices, and measured pesticide concentrations, the children could have been concurrently exposed to 34 distinct pesticides through their habitual diet. Weekly DE estimates for each analyzed food item are presented in Table 4.

Contributions to total DE differed substantially between raw and cooked food forms. In quantitative terms, raw food consumption accounted for 96% of total DE, with the remaining 4% attributable to cooked foods. The pesticide residues contributing the greatest estimated weekly DE, in descending order, were: pyrimethanil, boscalid, propamocarb, imidacloprid, chlorantraniliprole, thiophanate-methyl, pyraclostrobin, fluopyram, and carbendazim.

Residues detected in raw foods were predominantly non-neonicotinoid insecticides and acaricides, together with imidacloprid among the neonicotinoids, and the fungicides pyrimethanil and boscalid, both present at notable concentrations. In cooked foods, all detected neonicotinoids (imidacloprid, acetamiprid, clothianidin, dinotefuran, and thiamethoxam) accounted for the greatest proportional contribution, followed by pyraclostrobin, carbendazim, and spirodiclofen. Although imidacloprid was detected in cooked foods (upper-bound mean: 12.835 µg), its contribution from raw food consumption was approximately three times greater (upper-bound mean: 39.820 µg).

Among neonicotinoids, DE was markedly highest for imidacloprid, followed by acetamiprid, clothianidin, thiamethoxam, and dinotefuran. Exposure to neonicotinoids other than imidacloprid was attributable primarily to cooked food consumption.

## 4. Discussion

Of the 18 food types analyzed, fruits—including tomato—exhibited the highest pesticide residue loads: strawberry (13 compounds), raspberry (12), apple (10), grape (9), and tomato (9). These findings are consistent with reports from China and India documenting elevated multi-residue contamination in soft fruits and fruiting vegetables [30,42,43].

Seven of the 31 samples analyzed exceeded at least one MRL. Apple, tomato, and strawberry were found to contain carbendazim—a pesticide banned in the United States—although EU MRLs were not exceeded for these commodities. This finding underscores the need for international harmonization of regulatory limits to facilitate trade and, more critically, to protect human and ecological health. Carbendazim has been classified as a possible human carcinogen; given its serious toxicity and environmental persistence, its use in fruits and vegetables has been prohibited in multiple jurisdictions, including Australia, the United States, and the European Union [44,45]. The divergence among national regulatory frameworks constitutes a critical gap in the protection of vulnerable populations.

Regulatory monitoring systems such as the European Union’s Rapid Alert System for Food and Feed (RASFF) exemplify one mechanism through which pesticide residue exceedances are systematically tracked and communicated among member states and the European Commission, enabling rapid, coordinated responses to hazards detected in food, feed, and food contact materials [46]. Notifications related to pesticide residues constitute a substantial proportion of RASFF alerts, underscoring the ongoing relevance of residue monitoring even within a jurisdiction with comparatively stringent MRLs. Mexico currently lacks an equivalent rapid-alert infrastructure for pesticide residues in food; the absence of such a system may delay the identification and communication of non-compliant products, particularly for pesticide–commodity combinations for which no national MRL has been established, as observed for imidacloprid in guava and carbendazim in strawberry and apple in the present study. The development of a comparable rapid-alert mechanism, or Mexico’s closer alignment with RASFF-type reporting standards, could substantially strengthen the country’s capacity to detect and respond to pesticide residue non-compliance affecting vulnerable populations such as preschool children.

Apple, strawberry, cucumber, guava, and tomato were the principal contributors to total DE. Among these, apple was the dominant source of pyrimethanil (upper-bound mean: 336.502 µg/week; accounting for up to 82.8% of total DE). According to Chen et al. [47], pyrimethanil exerts adverse effects on animal development, hormonal homeostasis, and neural regeneration and has been linked to pathways implicated in Alzheimer’s disease, with pregnant women and infants identified as populations of heightened concern.

Strawberry was the primary contributor to boscalid DE (upper-bound mean: 237.6 µg/week; 95% of total boscalid DE). Although classified by regulatory agencies as having low acute toxicity, with the liver and thyroid identified as its principal target organs, boscalid acts as a succinate dehydrogenase inhibitor and has been shown to disrupt mitochondrial respiration and induce oxidative stress and early apoptosis in human hepatocytes [48]. This mitochondrial toxicity is of particular relevance given boscalid’s frequent co-occurrence with the strobilurin fungicide pyraclostrobin—as observed in the tomato samples analyzed in this study—since combined exposure of human hepatocytes to both compounds has been shown to potentiate mitochondrial dysfunction beyond the effect of either fungicide alone [49]. Cucumber was identified as the main source of propamocarb (99.8% of total DE; upper-bound mean: 230.760 µg/week). In rodent models, high-dose propamocarb exposure has been reported to induce metabolic disturbances mediated, at least in part, through alterations in gut microbiota composition and microbial metabolite profiles [50]. Propamocarb has additionally been reported to weakly stimulate aromatase activity in vitro, suggesting a low-potency endocrine-disrupting mechanism that could be amplified through simultaneous dietary co-exposure to other pesticides detected in this study [51].

Guava was the food with the highest imidacloprid DE contribution (61%; upper-bound mean: 69.6 µg/week), followed by tomato (32%; upper-bound mean: 36.739 µg/week). As the first neonicotinoid introduced commercially, imidacloprid is the compound with the most extensive literature documenting adverse effects on human health, encompassing cellular damage and mutagenicity [52], central nervous system effects [53,54], immune disruption [55], reproductive toxicity [56,57], hepatotoxicity [58,59], nephrotoxicity [55], gastrointestinal effects [55], and carcinogenic potential [60]. According to the dietary records, these fruits were predominantly consumed raw; only tomato was reported with a substantial cooked consumption component.

Among the quantifiable residues with the highest DE contributions, pyrimethanil and carbendazim were consistent with findings reported in China, where these compounds have been documented as high-frequency residues in fruits and vegetables [42]. Imidacloprid was the most frequently detected pesticide in the present study, in agreement with national data from Veracruz, Mexico [61] and international reports from China and India [24,30,41].

In cooked foods, all detected neonicotinoids contributed substantially to DE, a finding attributable to the marked thermostability of this insecticide class. Non-neonicotinoid compounds including pyraclostrobin and spirodiclofen also exhibited notable contributions in cooked food matrices. The influence of thermal processing on final pesticide concentrations is mediated by multiple variables, including compound transfer kinetics between food peel and pulp. For instance, boscalid has been reported to remain predominantly in the skin fraction, whereas pirimicarb may transfer to the pulp at rates of up to 83% [34]. The type of thermal process also exerts a significant influence: boiling of tomato has been shown to degrade boscalid almost entirely (PF = 0.03), whereas neonicotinoids such as thiamethoxam and imidacloprid retain concentrations corresponding to PF values of approximately 0.58 [32,35]. Notably, acetamiprid concentrations have been reported to increase during certain drying processes, while other pesticides are concurrently degraded [34,35].

To the authors’ knowledge, no studies have yet been published on the additivity and combined effects of the specific multi-pesticide mixtures documented in the present study and their implications for health risk in pediatric populations.

Several methodological limitations should be considered when interpreting these exposure estimates. This was a cross-sectional study conducted in a single preschool community, with food samples collected by convenience sampling from the retail sites reported by the 23 participating families, rather than through a randomized, regionally representative design. Several food items—including guava, avocado, and both maize varieties—were represented by only one sample; given that pesticide use and residue levels can vary across plots, seasons, and harvests, single-sample results should be regarded as indicative rather than robust point estimates. Sampling was also limited to a single period, without seasonal replication, so temporal variation in application timing and residue degradation was not captured. These estimates should therefore be understood as an exploratory, community-level characterization of dietary pesticide exposure rather than a generalizable regional risk assessment.

## 5. Conclusions

The present study documents multi-residue pesticide contamination in staple foods consumed by preschool children residing in farming areas of western Mexico. It further estimates pesticide persistence throughout food processing and evaluates cumulative DE, providing toxicologically relevant findings with significant implications for pediatric public health.

Of the 31 food samples analyzed by LC/ESI-MS/MS, 87% contained at least one detectable pesticide residue, comprising 34 compounds from diverse chemical families, including five neonicotinoids (imidacloprid, acetamiprid, clothianidin, dinotefuran, and thiamethoxam). The distribution of pesticide contamination was not homogeneous across food matrices. Fruits represented the commodity group with the highest number of simultaneous residues, with up to 13 pesticides detected in a single strawberry sample and 12 in raspberry, followed by apple (10), grape (9), and tomato (9).

Seven samples exceeded MRLs established by the EU, US, and/or Mexico, reflecting both non-compliance with food safety standards and pronounced regulatory disparity among jurisdictions. Of particular concern is the detection of carbendazim —a possible human carcinogen banned in the US—in strawberry, apple, and tomato, in the absence of national MRLs for several of these matrices in Mexico, underscoring a critical gap in the protection of vulnerable populations.

Weekly DE was highest for pyrimethanil, boscalid, propamocarb, and imidacloprid, with raw food consumption accounting for 96% of total exposure and the remaining 4% attributable to cooked foods, underscoring the importance of culinary practices in modulating pesticide intake. Among neonicotinoids, imidacloprid contributed most to detection frequency and the greatest contribution to total DE, in both raw and cooked food forms. The notable thermostability of neonicotinoids, evidenced by processing factors ranging from 0.4 to 0.99, indicates that cooking does not substantially reduce their residue loads, thereby increasing the potential for chronic exposure through prepared foods.

Current regulatory reference doses and risk assessments evaluate neonicotinoids on a single-compound basis and therefore do not capture the multi-pesticide co-exposure documented in this study. This limitation represents both the primary constraint of current regulatory frameworks and the major toxicological uncertainty of the present study. The multi-pesticide co-exposure to up to 13 distinct pesticides within a single food commodity, with heterogeneous mechanisms of action encompassing acetylcholinesterase inhibition, endocrine disruption, neurotoxicity, hepatotoxicity, and immunotoxicity, constitutes a cumulative risk scenario whose magnitude cannot be adequately assessed using conventional single-compound risk assessment paradigms. The scientific evidence on mixture toxicity of pesticides in pediatric populations remains limited, representing a critical gap in dietary risk assessment for agroindustrial zones.

These findings should be interpreted considering the study’s cross-sectional design, the small cohort of 23 families, and the convenience-based sampling of 31 food items, several represented by a single measurement and without seasonal replication, which limit generalization of the exposure estimates to the broader regional child population. Nonetheless, to the authors’ knowledge, this is the first study to characterize dietary exposure to neonicotinoids and other pesticides among preschool children in this agroindustrial region and one of the first in Mexico, providing an early indicator that these children are repeatedly exposed, through their habitual diet, to multiple pesticides with documented adverse effects on human health. Longitudinal studies are needed to determine whether this repeated exposure translates into chronic exposure over time.

In conclusion, the present findings demonstrate that preschool children residing in areas where more technology is applied and more pesticides are used in agricultural production systems are repeatedly and simultaneously exposed to residues of multiple pesticides through their habitual diet, with commonly consumed items such as tomato, apple, strawberry, cucumber, and guava constituting the primary exposure sources. The magnitude and chemical diversity of this multi-pesticide co-exposure and its associated toxicological complexity urgently warrant longitudinal epidemiological studies to evaluate cumulative impacts on child health, together with revision of national regulatory frameworks to incorporate pesticide mixture assessment criteria, harmonization of MRLs across jurisdictions, and strengthening of food safety monitoring programs specifically targeted at vulnerable population groups. Such monitoring programs could be informed by rapid-alert models such as the EU’s RASFF system, which could serve as a reference framework for developing similar early-warning mechanisms in Mexico.

## Figures and Tables

**Table 2:** Pesticides concentration in vegetal matrices exceeding the Maximum Residue Levels (MRLs) for any standard. EU: European Union; USA: United States of America; MX: Mexico. WI: without information; PROH: prohibited; Shaded in gray: the exceeded rule [39,40,41].

Pesticide	Food Matrix	Sample Number	Concentration (mg/kg)	EU MRLs ^1^ (mg/kg)	USA MRLs (mg/kg)	MX MRLs (mg/kg)
Imidacloprid	Guava	1	0.29	0.05	1.0	WI
Spirodiclofen	Chayote	1	0.034	0.02	NE	WI
Boscalid	Strawberry	1	1.8	6.0	4.5	1.2
Fluoxastrobin	Avocado	1	0.094	0.01	1.0	1.0
Thiophanate-Methyl	Strawberry	1	0.17	0.1	7.0	5.0
Carbendazim	Strawberry	2	0.023/0.088	0.1	PROH	WI
	Tomato	1	0.0067	0.3	PROH	0.5
	Apple	1	0.0063	0.2	PROH	WI

^1^ Maximum Residue Levels (MRLs) in mg/kg of food.

**Table 3:** Average Weekly Consumption of Foods by Type of Process (Raw Consumption, Cooked Consumption and the Total), According to the Dietary Records Completed for Mexican Children.

Type of Food	Food Matrix	Raw Consumption (g)		Cooked Consumption (g)		Total Consumption (g)	
		Number of Children	Consumption Mean (sd)	Number of Children	Consumption Mean (sd)	Number of Children	Consumption Mean (sd)
Cereal	Corn on cob	0	0.000	18	72.658 (72.276)	18	72.653 (72.276)
	Corn tortilla	0	0.000	23	475.186 (236.066)	23	475.187 (236.066)
Fruit	Raspberry	7	64.785(47.835)	0	0.000	7	64.786 (47.836)
	Strawberry	10	88.421 (84.470)	0	0.000	10	88.421 (84.470)
	Guava	10	112.905 (93.230)	0	0.000	10	112.905 (93.231)
	Tangerine	4	405.25 (226.870)	0	0.000	4	405.25 (226.871)
	Apple	21	395.419 (216.185)	0	0.000	21	395.419 (216.185)
	Orange	17	473.888 (486.205)	0	0.000	17	473.888 (486.205)
	Banana	20	403.131 (426.907)	2	150 (70.711)	20	418.131 (427.130)
	Green grape	6	151.666 (112.234)	0	0.000	6	151.667 (112.235)
Tuber	Potato	0	0.000	23	124.576 (102.9)	23	124.576 (102.900)
Vegetables	Avocado	18	34.666 (24.049)	0	0.000	18	34.667 (24.049)
	Onion	15	15.033 (14.770)	23	109.870 (53.732)	23	119.675 (53.583)
	Chayote	0	0.000	18	151.771 (96.590)	18	151.771 (96.590)
	Tomato	17	50.529 (34.092)	23	253.858 (122.202)	23	291.206 (122.457)
	Cucumber	17	186.529 (185.838)	0	0.000	17	186.529 (185.838)
Legume	Bean	0	0.000	21	120.191 (71.665)	21	120.191 (71.665)

**Table 4:** Weekly Dietary Exposure to Pesticides through Food Consumption, Classified by Raw Foods, Cooked Food and Total Exposure, in Mexican Children. Two Scenarios were Considered for Calculating Pesticide Concentration: Lower-Bound and Upper-Bound Limits in Each Type of Food Processing.

	Exposure by Raw Foods (µg)		Exposure by Cooked Foods (µg)		Total Weekly Dietetic Exposure (µg)	
Pesticide	Lower Limits	Upper Limits	Lower Limits	Upper Limits	Lower Limits	Upper Limits
	Mean	Mean	Mean	Mean	Mean	Mean
Pyrimethanil	406.937	408.340	0.000	0.375	406.937	408.715
Boscalid	84.931	85.184	0.221	0.236	85.152	85.420
Propamocarb	67.151	67.353	0.000	0.000	67.151	67.353
Imidacloprid	39.439	39.820	12.586	12.835	52.025	52.655
Chlorantraniliprole	10.920	11.780	0.000	0.000	10.920	11.780
Thiophanate-Methyl	7.516	7.516	0.000	0.000	7.516	7.516
Pyraclostrobin	3.673	3.877	5.315	5.395	8.988	9.272
Carbendazim	6.322	6.502	0.680	1.546	7.003	8.048
Fluopyram	7.887	8.084	0.000	0.000	7.887	8.084
Hexythiazox	6.900	6.900	0.000	0.000	6.900	6.900
Acetamiprid	1.121	1.534	3.644	3.732	4.766	5.267
Fenazaquin	2.034	2.034	0.000	0.000	2.034	2.034
Fenhexamid	2.973	2.973	0.000	0.000	2.973	2.973
Clothianidin	0.379	0.897	1.104	1.493	1.483	2.390
Fluoxastrobin	1.629	1.629	0.000	0.111	1.629	1.740
Thiamethoxam	0.505	0.658	1.472	1.472	1.978	2.131
Spirodiclofen	0.000	0.000	1.213	1.213	1.213	1.213
Flutriafol	0.000	1.186	0.000	0.000	0.000	1.186
Fenpyroximate	0.376	0.529	0.000	0.000	0.376	0.529
Bifenazate	0.597	0.597	0.000	0.000	0.597	0.597
Spirotetramat	0.000	0.607	0.000	0.049	0.000	0.656
Spinetoram	0.000	0.585	0.000	0.000	0.000	0.585
Methoxyfenozide	0.000	0.152	0.174	0.174	0.174	0.326
Dimethoate	0.000	0.299	0.000	0.000	0.000	0.299
Carbaryl	0.000	0.395	0.000	0.000	0.000	0.395
Thiabendazole	0.243	0.243	0.000	0.000	0.243	0.243
Dinotefuran	0.056	0.071	0.112	0.156	0.167	0.226
Novaluron	0.000	0.101	0.000	0.075	0.000	0.177
Spinosyn A	0.000	0.097	0.000	0.000	0.000	0.097
Formetanate	0.000	0.065	0.000	0.000	0.000	0.065
Spinosyn D	0.000	0.022	0.000	0.000	0.000	0.022
Forchlorfenuron	0.000	0.032	0.000	0.000	0.000	0.032
Methomyl	0.000	0.000	0.000	0.012	0.000	0.012
Mandipropamid	0.000	0.000	0.000	0.007	0.000	0.007

Note: All estimations are calculated in µg.

## Data Availability

The data presented in this study are openly available in https://www.riudg.udg.mx/handle/20.500.12104/96296. (accessed on 20 May 2026). Data will be made available from the corresponding author upon reasonable request.

## Abbreviations

The following abbreviations are used in this manuscript:

DE	Dietary Exposure
MRLs	Maximum Residue Limits
MQL	Method Quantitation Limit
MDL	Method Detection Limit
PF	Processing Factor
